# *Berberis* Plants—Drifting from Farm to Food Applications, Phytotherapy, and Phytopharmacology

**DOI:** 10.3390/foods8100522

**Published:** 2019-10-22

**Authors:** Bahare Salehi, Zeliha Selamoglu, Bilge Sener, Mehtap Kilic, Arun Kumar Jugran, Nunziatina de Tommasi, Chiara Sinisgalli, Luigi Milella, Jovana Rajkovic, Maria Flaviana B. Morais-Braga, Camila F. Bezerra, Janaína E. Rocha, Henrique D.M. Coutinho, Adedayo Oluwaseun Ademiluyi, Zabta Khan Shinwari, Sohail Ahmad Jan, Ebru Erol, Zulfiqar Ali, Elise Adrian Ostrander, Javad Sharifi-Rad, María de la Luz Cádiz-Gurrea, Yasaman Taheri, Miquel Martorell, Antonio Segura-Carretero, William C. Cho

**Affiliations:** 1 Student Research Committee, School of Medicine, Bam University of Medical Sciences, Bam 44340847, Iran; bahar.salehi007@gmail.com; 2 Department of Medical Biology, Faculty of Medicine, Nigde Ömer Halisdemir University, Campus, 51240 Nigde, Turkey; zselamoglu@ohu.edu.tr; 3 Department of Pharmacognosy, Faculty of Pharmacy, Gazi University, 06330 Ankara, Turkey; bilgesener11@gmail.com (B.S.); klcmehtap89@gmail.com (M.K.); 4 G.B. Pant National Institute of Himalayan Environment and Sustainable Development, Garhwal Regional Centre, Srinagar 246174, Uttarakhand, India; arunjugran@gmail.com; 5 Department of Pharmacy, University of Salerno, Via Giovanni Paolo II 132, 84084 Fisciano, Italy; detommasi@unisa.it; 6 Department of Science, University of Basilicata, Viale dell’Ateneo Lucano 10, 85100 Potenza, Italy; chiara.sinisgalli@gmail.com (C.S.); luigi.milella@unibas.it (L.M.); 7 Institute of Pharmacology, Clinical Pharmacology and Toxicology, Medical Faculty, University of Belgrade, 11129 Belgrade, Serbia; jolarajkovic@yahoo.com; 8 Laboratory of Applied Micology of Cariri—LMAC, Regional University of Cariri—URCA, Crato 63105-000, CE, Brazil; flavianamoraisb@yahoo.com.br (M.F.B.M.-B.); camilawasidi@gmail.com (C.F.B.); 9 Laboratory of Microbiology and Molecular Biology—LMBM, Regional University of Cariri—URCA, Crato 63105-000, CE, Brazil; janainaesmeraldo@gmail.com (J.E.R.);; 10 Functional Foods, Nutraceuticals and Phytomedicine Unit, Department of Biochemistry, Federal University of Technology, Akure 340252, Nigeria; AOADEMILUYI@futa.edu.ng; 11 Department of Biotechnology, Quaid-i-Azam University, Islamabad 45320, Pakistan; shinwari2008@gmail.com; 12 Department of Biotechnology, Hazara University Mansehra, Khyber Pakhtunkhwa 21120, Pakistan; sjan.parc@gmail.com; 13 Department of Chemistry, Faculty of Science, Mugla Sitki Kocman University, Mugla 48121, Turkey; e.ebrusimya@gmail.com; 14 National Center for Natural Products Research, School of Pharmacy, University of Mississippi, Oxford, MS 38677, USA; zulfiqar@olemiss.edu; 15 Medical Illustration, Kendall College of Art and Design, Ferris State University, Grand Rapids, MI 49501, USA; eliseadrianostrander@gmail.com; 16 Department of Pharmacology, Faculty of Medicine, Jiroft University of Medical Sciences, Jiroft 7861756447, Iran; 17 Department of Analytical Chemistry, Faculty of Sciences, University of Granada, Avda. Fuentenueva s/n, 18071 Granada, Spainansegura@ugr.es (A.S.-C.); 18 Research and Development Functional Food Centre (CIDAF), Bioregión Building, Health Science Technological Park, Avenida del Conocimiento s/n, 188016 Granada, Spain; 19 Phytochemistry Research Center, Shahid Beheshti University of Medical Sciences, Tehran 1991953381, Iran; taaheri.yasaman@gmail.com; 20 Department of Pharmacology and Toxicology, School of Pharmacy, Shahid Beheshti University of Medical Sciences, Tehran 11369, Iran; 21 Department of Nutrition and Dietetics, Faculty of Pharmacy, University of Concepcion, Concepcion 4070386, Chile; martorellpons@gmail.com; 22 Unidad de Desarrollo Tecnológico, Universidad de Concepción UDT, Concepcion 4070386, Chile; 23 Department of Clinical Oncology, Queen Elizabeth Hospital, 30 Gascoigne Road, Hong Kong, China

**Keywords:** *Berberis*, food preservative, alkaloid, antioxidant, human health

## Abstract

The genus *Berberis* includes about 500 different species and commonly grown in Europe, the United States, South Asia, and some northern areas of Iran and Pakistan. Leaves and fruits can be prepared as food flavorings, juices, and teas. Phytochemical analysis of these species has reported alkaloids, tannins, phenolic compounds and oleanolic acid, among others. Moreover, *p*-cymene, limonene and ocimene as major compounds in essential oils were found by gas chromatography. *Berberis* is an important group of the plants having enormous potential in the food and pharmaceutical industry, since they possess several properties, including antioxidant, antimicrobial, anticancer activities. Here we would like to review the biological properties of the phytoconstituents of this genus. We emphasize the cultivation control in order to obtain the main bioactive compounds, the antioxidant and antimicrobial properties in order to apply them for food preservation and for treating several diseases, such as cancer, diabetes or Alzheimer. However, further study is needed to confirm the biological efficacy as well as, the toxicity.

## 1. Introduction

*Berberis* spp. are shrubs in the family *Berberidaceae*, native to central and southern Europe, western Asia, as well as northwest Africa [1]. About 500 species of these plants are found in most areas of central and southern Europe, the north-eastern region of United States, and Asia (including the northern area of Pakistan [2] and Iran [3]). The genus *Berberis* consists of spiny deciduous evergreen shrubs which are characterized by yellow wood and flowers [2], dimorphic long and short shoots (1–2 mm). Some *Berberis* fruits are small oblong berries 7–10 mm long and 3–5 mm broad and turn blue or red upon ripening during the late summer or autumn [1].

*Berberis* species are mainly consumed fresh, dried and used in juice production [4]. The fruits are very popular, known as *zereshk* in Iran where they are commonly used for cooking and in jam production, thus, encouraging the production of fresh edible seedless barberries fruits reaching about 22,000 tons per annum [5]. The fruits are also processed into beverages, drinks, syrups, candy and other confectionary products which are popular Iran. Furthermore, the leaves and fruits have also found applications in the production of food flavorings and teas. *Berberis* are popular due to their nutritional importance; however, they have found most usefulness in folk and traditional medicine where various parts, including roots, bark, leaves and fruits serve as major ingredients of herbal remedies in Ayurvedic, Iranian and Chinese medicine dating back at least 3000 years [6]. Currently, this species flower is popularly used amongst Tibetan speaking population in areas, such as Litang, China [7].

The effect of cold-pressed filtered oil of *Berberis* spp. seeds in delaying soybean oil oxidation in comparison to commercial antioxidants were carried out, and the study reported that *Berberis* oil contributed to oxidative stability of soybean oil comparably to commercial antioxidants [8]. Antioxidant and antibacterial activity of water extract of barberry has suggested their possible application as preservatives in food industries [9].

Isoquinoline alkaloids are the major bioactive constituents in *Berberis* [10]. Protoberberines and bisbenzyl-isoquinoline alkaloids, such as berbamine, tetrandrine and chondocurine, which have been known for their anti-inflammatory and immunosuppressive properties, have been detected by phytochemical analysis of the root and stem back extracts of *B. vulgaris*. Berberine (an isoquinoline alkaloid) and berbamine are the most abundant phytochemicals of *Berberis* species [2]. The fruits contain a high amount of alkaloids, tannins, phenolic compounds and oleanolic acid [3,11], gum, pectin, oleoresins, organic acids, anthocyanins and carotenoids. In addition, palmitine [10], stigmasterol and its glycoside [12] have all been detected in various species of the *Berberis* plant.

Some *Berberis* fruits have been employed in the treatment of guts [13] kidney stones [14] and liver [15] and gall bladder [10] conditions. The root bark and stem of the *Berberis* have found usage as a diuretic, febrifuge, cathartic and antiseptic. Furthermore, preparations of the stem and root bark have been used to treat mouth and stomach ulcers [16]. Several parts of the plant have been reported to possess astringent and antiseptic properties, while the stem bark and flowers were found to be anti-rheumatic [17]. The alkaloid rich root bark of the plant has also been used as purgative and treatment for both diarrhea and rheumatism [18]. The berberine-rich rhizomes of *Berberis* species possess marked antibacterial and antitumor properties, with reported efficacies in treatment of various eye conditions [10,19]. Furthermore, the anti-inflammatory activity of berberine has been extensively studied amongst other pharmacological actions [10,20].

Berberine sulphate which is an alkaloid extracted from the roots and bark of various *Berberis* spp. Have been reported to possess antibacterial, antifungal and antiprotozoal activities. Reported the bacteriostatic activity of berberine against streptococci, and that the sub-minimum inhibitory concentrations (MICs) of the compound blocked the adherence of streptococci to host cells, immobilized fibronectin, and hexadecane in epithelial cells [21]. Furthermore, blood glucose and lipid regulatory properties of *Berberis* have been demonstrated [3,22,23,24]; and this was due to berberine-induced improvement in insulin sensitivity through regulation of adipokine secretion [25,26,27]. Effectiveness of *Berberis* species in the maintenance of heart health has been demonstrated in their ability to improve hypertension, ischemic heart disease, cardiac arrhythmias and cardiomyopathy [2,28].

The health-promoting effect of *Berberis* spp. cannot be overemphasized, as well as its popularity; however, this is restricted to central and southern Europe, western Asia, as well as northwest Africa. Hence, efforts should be geared towards making the *Berberis* plant also available to other regions of the world. Furthermore, most studies on *Berberis* spp. have been on berberine; therefore, efforts should be made towards researching possible therapeutic benefits of all other important phytoconstituents of the plant. Furthermore, the synergistic or additive effect of these phytoconstituents should be studied so as to elucidate the complex molecular interaction amongst various phytochemicals leading to the observed therapeutic properties. In addition, the modulatory effect of the plant/plant materials on gene expression should be prioritized.

The aim of this review is to provide a detailed overview to the cultivation of *Berberis* species, in-depth insight on the biological properties of the phytoconstituents of this genus, regarding its food preservative applications, antimicrobial, antioxidant and anticancer effects, and lastly, special emphasis to its clinical effectiveness in humans. The present work was performed by consulting the database of PubMed, Web of Science, Embase, and Google Scholar (as a search engine) to retrieve the most updated articles on the topic under investigation (phytochemicals and biological activities of *Berberis* species). The strategy of the search included the use of the following keywords: “*Berberis*” or “barberry” and “cultivation” or “essential oil” or “antimicrobial” or “food preservative” or “antioxidant” or “anticancer”. Authors carefully examined articles and for the review, prioritizing the articles published from 2013 to 2018 [29]. Only English articles having full text were considered.

## 2. Cultivation of *Berberis* Plants

**Figure 1:**
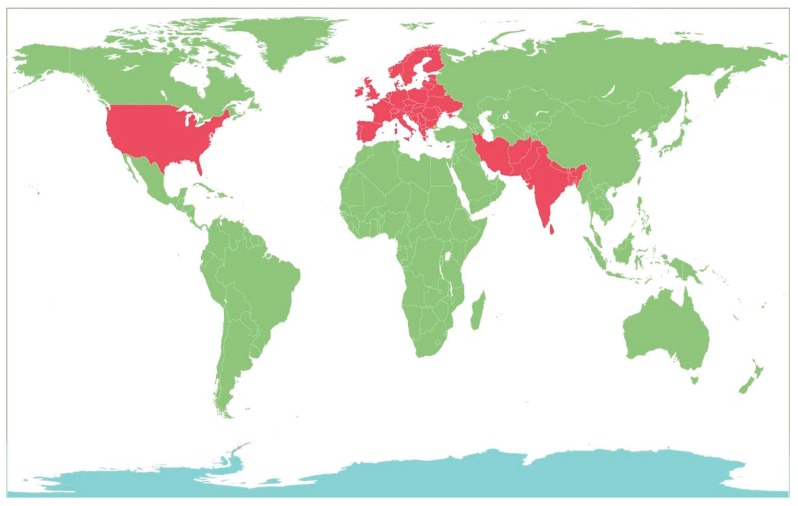
The areas where the *Berberis* plants are commonly grown (shown in red).

The genus *Berberis* include about 500 different species and commonly grown in Europe, United States, South Asia and some northern areas of Iran and Pakistan (Figure 1) [11,30]. In Pakistan, majority of *Berberis* species are found in high mountains (1400 m–3500 m above sea level). In Iran, five *Berberis* species are present, including two important species, i.e., *B. orthobotrys* and *B. khorassanica*, which are grown in eastern, northern and southern regions of Iran [31]. Other important local species *zereshk* is also widely cultivated in the South-Khorasan province of Iran [32]. In Iran, 11,000 hectares of area is under cultivation of common barberry (*B. vulgaris*) species, and one of the most leading producers of barberry fruit in the world. Annually, Iran produces more than 10,000 tons of dried barberry fruits, while maximum production comes from the Southern Khorasan. More than 97% of the area located near Ghaenat County and Southern Khorasan province is cultivated with common barberry that produces 95% of fruit in Iran [33]. *B. vulgaris* is gathered from the wild in Eastern Europe in countries like Poland [34,35]. In addition, it is known as *K’otsakhuri* in Georgia, where it is grown and collected from the southeast forests of the country [36]. The yield-related important traits of many *Berberis* species are significantly affected by environmental factors, biotic stresses, seasonal variations, the climatic condition of an area, planting and harvesting date or methods, irrigation source, fruit processing and storage methods, etc.

The fruit remains a vital part, producing many important secondary metabolites and used in pharmaceutical and food industries. In this section, we have highlighted the cultivation of different *Berberis* species, its status and various factors affecting its cultivation. Common barberry is a native plant in Asia’s western and middle mountains and non-native to North America [37]. The hybrid species *Berberis* × *ottawaensis* are widely cultivated in Europe and North America [38]. The European settlers introduced common barberry to New England, and used it as a source of medicine, food, and for other aesthetic purposes [37,39]. The colonists of New England determined its cultivation spread *Puccinia graminis* fungus, causing wheat rust and important reduction of its crop [40].

The seedless type (*B. vulgaris* var. *asperma*) is commonly cultivated in the southern parts of the Khorasan province of Iran for domestic purposes [41]. *B. lyceum* represents a native species of Nepal and distributed in the temperate and subtropical regions of the world, including some part of Australia. It is distributed from Kashmir to Uttaranchal North-western Himalayas [42]. Sixteen species, and some varieties of barberry, were found in the Boaxing country, situated on the Eastern side of Hengduan Mountains in Sichuan Province, Southwest China [43]. The Japanese barberry is invasive in twenty different states of the world and five other provinces of Canada [44]. According to Reference [45] there are 21 *Berberis* species in Nepal, including two new species *Berberis pendryi* Bh. Adhikari and *Berberis karnaliensis* Bh. Adhikari. *Berberis crataegina* DC. is commonly grown in Turkey, Asia and European regions. The fruit are locally known as “karamuk” and “kadıntuzluğu” in Turkey and used as traditional medicine [46].

In Pakistan, twenty species and six subspecies of *Berberis* have been reported, and the majority of these is growing in northern parts of the country [47]. The other dominant species of *Berberis* is *B. aristata*, grown in Nepal, Pakistan and India. In Pakistan, it mainly cultivated in the Hazara division of Khyber Pakhtunkhwa (KP), and Azad Jamu and Kashmir (AJK) regions. In AJK it is locally named s*umbal* and commonly known as *daruharidra* [48]. *B. lyceum* is another key species, highly distributed in different Asian countries, including, Afghanistan, Pakistan, Nepal, India and Bangladesh. In Pakistan, it grows in different areas of KP, Punjab and Baluchistan [49]. The fruit part locally called *kashmal*, is used as a source of food, and for preparing the sauce in Himalayan regions of Jammu and Kashmir, and Himachal Pradesh [50,51].

The yield of barberry plants depends on various factors like managing operations, size and age of shrub and date and method of harvesting [52,53]. Proper harvesting method at a suitable time is one of the key steps in berry yield production because the shrubs include maximum spines in shoot part and also the fruit peel is so thin. The harvesting date plays a vital role to gain maximum yield with high quality. The local farmers set some useful sensory parameters for starting the harvest of the crop. These parameters include change of fruit color from bright red to dark red, tissue softening, the concentration of contents, and reducing sourness in fruit, etc. [11]. The optimum time for barberry harvesting in the autumn cold season, when the berries ripen. In this stage, the fruit gains dark red color due to the presence of high anthocyanin content, sweetness increases, while the berberine and sourness are reduced [53,54,55,56,57]. In different regions of North America and Western Europe, the common Barberry ripens in the month of August or September [58,59]. While the seeds can be mature in October [60]. The berries of common barberry remain with the stem through winter [61]. However, the delay in harvesting from 10 September to 13 November increases the anthocyanin content about 2.5 times [53]. It may also increase the yield and quality, but too much delaying may lead to early autumn chilling injury to plant. For Iranian seedless barberry 170 days after the flowering is an optimum date for harvesting [56]. Fruit maturation and development vary in different geographic regions. So, it is important to optimize a suitable harvesting date for each region.

## 3. *Berberis* Plants Essential Oils and Phytochemical Composition

Essential oils (EO) are volatile, complex natural compounds, which formed in aromatic plants as secondary metabolites. They are used in pharmaceutical, agricultural, and food industries, as well as are associated with antibacterial, anti-inflammatory, antioxidant, and insecticidal potential [62,63,64].

**Figure 2:**
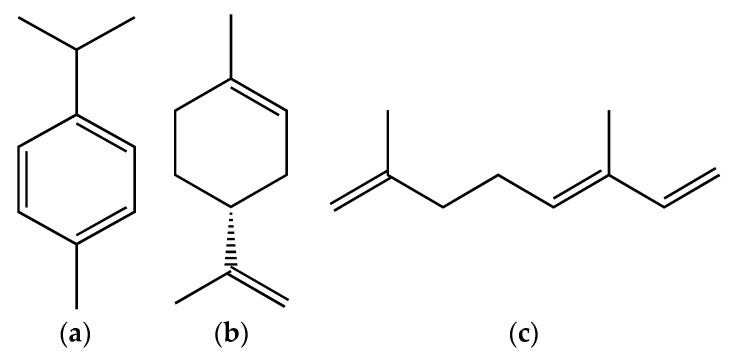
Major compounds of the essential oils (EOs) of *Berberis vulgaris* leaves and flowers. (**a**) *p*-cymene; (**b**) limonene; (**c**) ocimene.

The gas chromatography coupled to mass spectrometry (GC-MS) analysis of various parts of *B. vulgaris* revealed that benzaldehyde, benzyl alcohol, 1-hexanol and I-2-hexenal [65] were major compounds of the EOs from fruit, while *p*-cymene, limonene and ocimene were identified as major compounds of the EOs (Figure 2) from leaves and flowers [66].

Turkish *B. crataegina* fruit berry has 22 volatile compounds which are aldehydes had the highest concentration (5382 μg/kg), followed by alcohols (2487 μg/kg) and lactone (2422 μg/kg).

Major volatile compounds of the *B. crataegina* fruit are *γ*-butyrolactone, 3-hexanal and 2,6-dimethylphenol. Moreover, the olfactometric analysis of dry *B. crataegina* resulted eight aroma active compounds [67].

EOs of the roots of *B. integerrima* were analyzed by using modified microwave-assisted hydrodistillation (MAHD). Chemical diversity of 10 and 18 compounds were obtained from MAHD, MAHD with modified anyl, and with modified phenyl magnetic nanoparticles, the yields of the EOs were 0.16, 0.61 and 0.71 *w/w* %, respectively. Hexadecanoic acid was identified as a major compound for MAHD and modified MAHD methods [68].

**Figure 3:**
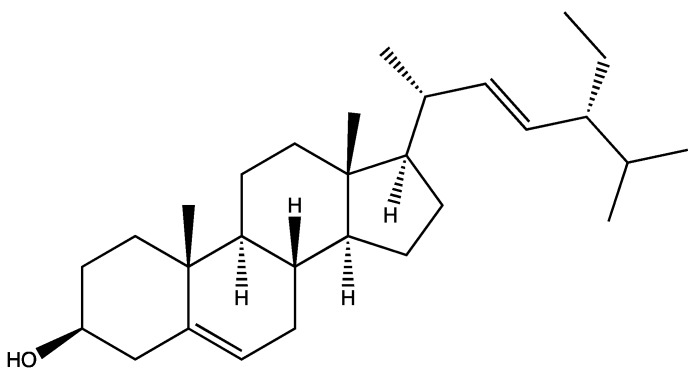
Stigmasterol.

Moreover, the GC/MS study on hexane extracts of the *B. aetnensis* and *B. libanotica* roots was showed that *B. aetnensis* have twenty-six and *B. libanotica* have thirty-seven non-polar compounds. Stigmasterol (Figure 3) is the major compound of both species [69].

On the other hand, alkaloids (Table 1) represent the main compounds in *Berberis* species, and many of them have been identified by different spectroscopic techniques previously mentioned. The most known are berberine, berbamine, palmitine, jatrorrhizine, and isotetrandrine. They are located mainly in the cortical tissues of the roots and stems and have important biological activities. In fact, in vitro and in vivo anti-proliferative and anti-metastatic effects on various types of cancers have been reported for different alkaloids. These compounds, such as vinblastine, have already used as anticancer drugs [3].

## 4. Food Preservative Applications of *Berberis* Plants

Food preservation is the most vital issue in food industries to ensure food safety for a longer period. Basically, the process of food preservation depends on the growth inhibition of undesirable microorganisms. Use of chemical agents with antimicrobial activity is commonly used a traditional method for food preservation [70]. However, antimicrobial agents also gain momentum, due to their fewer side effects and compatibility with the human body. Further, synthetic antimicrobials and their toxicological safety as food additives needed to be ensured by regulatory authorities. Moreover, processed foods with natural preservatives have great demand and considered safer and beneficial for public health [71]. The naturally occurring compounds demonstrated antimicrobial activity in foods as natural ingredients and can be used as additives to other foods.

*Berberis* is an important plants having enormous potential in the food industry. However, only a few reports are available on the direct application of these plants in food products. For example, seed oil and fruit extracts of *B. crataegina* were supplementing into chitosan matrix for preparation of a chitosan-based edible film. The films produced have been analyzed for the physiochemical and biological activities. Results showed that chitosan-fruit extract film exhibited higher thermal stability, antimicrobial, antioxidant, and anti-quorum sensing activity as compared to other films. Furthermore, the addition of *B. crataegina* seed oil and fruit extract into the chitosan film create a mark reduction in the UV-vis transmittance but improve the tensile strength. Likewise, hydrophobicity of the chitosan-seed oil film was found to be higher than chitosan-control film, while chitosan-fruit extract film displayed slightly lower hydrophobicity than chitosan film. These results indicated that chitosan-fruit extract film of *B. crataegina* fruit extract could be used as an effective ingredient for the production of the edible film with increased physicochemical and biological properties [72].

A list of the antimicrobial potential of the *Berberis* species evaluated across the globe is provided which support the use of *Berberis* species in food preservation (Table 2).

## 5. Antioxidant Activities of *Berberis* Plants (In Vitro and In Vivo)

Free radicals ubiquitous in the environment affect human health by oxidative stress-induced damage. Finding exogenous sources with antioxidant activity is necessary in order to support the organism against the actions of free radicals. The fruits of most plants from Berberidaceae family have a sour taste which is due mainly to the presence of ascorbic acid or vitamin C. The vitamins and antioxidant compounds in barberry plant might be useful for treating diseases [109].

The antioxidant effect of *B. vulgaris* on oxidative systems, such as liver cells oxidation, red blood cells haemolysis, and haemoglobin non-enzymatic glycosylation was demonstrated, and the highest inhibitory effect was exerted on glycosylation. The extracts of *B. vulgaris* was the most promising as antioxidants, as well as anti-inflammatory and acetylcholinesterase (AChE) inhibitors. The capacity of *B. vulgaris* for scavenging 1,1-diphenyl-2-picrylhydrazyl (DPPH) and 2,2′-azino-bis(3-ethylbenzothiazoline-6-sulphonic acid) (ABTS), the inhibitions of lipoxygenase and AChE are mainly due to the phenol and flavonoid contents [110].

*B. vulgaris* root extract was evaluated for the alleviation of oxidative stress by using female Japanese quails. Moreover, *B. vulgaris* root extract exerted antioxidant effects through inhibiting NF-kB, which was activated and suppressed in the heat stress environment [111]. The antioxidant potential of 50% aqueous ethanolic root extract of *B. aristata* was examined on antioxidant enzymes of the liver in diabetic rats, along with its safety parameters. The root extract of *B. aristata* has strong potential to decrease oxidative stress [112].

Significant antioxidant effects, mainly on ABTS, hydroxyl radicals and DPPH, have been reported to berberine hydrochloride. The relationship among diabetes mellitus and the increase of formation of free radicals and a decrease in antioxidant potential is well known. Since berberine hydrochloride has significant radicals scavenging and protective effects against *β*-cell damage and antioxidant of the pancreas in diabetes mellitus, it seems reasonable that antioxidants can play an important role in the improvement of diabetes and in screening the novel treatment drug of diabetes mellitus [113].

The extracts from the inner stem bark of *B. vulgaris* exhibited high antioxidant activity, and most of the identified compounds were isoquinoline alkaloids. The values were higher than the standard antioxidant compounds (vitamin C and butylated hydroxytoluene (BHT)) [114].

It was widely investigated the composition of the major anthocyanins and the antioxidant activities of the fruit of *B. heteropoda*. The high anthocyanins content indicated that this fruit could be considered as an excellent source of natural colorants and a functional food that benefits human health [115]. Regarding the alkaloid extract of *B. aetnensis* roots, it is also possessed antioxidant properties, and all results are in agreement with other reports on the alkaloids from the roots of *Berberis* species [116].

The findings suggested that *B. vulgaris* fruits have an important potential for their antioxidant activities depends on the content of phenolic compounds and organic acids [117].

## 6. Anticancer Activities of *Berberis* Plants (In Vitro and In Vivo)

Cancer is the third leading cause of death worldwide, preceded by cardiovascular and infectious diseases. In medical science, there is need for effective and acceptable cancer therapeutics agents that are non-toxic, highly efficacious against multiple cancers, cost effective, and acceptable by human population [118]. The interest in natural products has increased because they are less toxic to normal cells, and they reduce side effects and drug resistance observed in synthetic drugs.

In India, medicinal plants have been used for treating disease since ancient times. Many of these belong to the genus *Berberis*. The fruit of *B. vulgaris* is rich in polyphenols, vitamins, proteins, ascorbic acid, and anthocyanin, which are important for human health. Moreover, they are rich in alkaloids which promote anticancer activity. In particular in hepatic cancer cell (Hepg2) ethanol extract of the fruit of *B. vulgaris* reduces cell vitality and promotes the selective increase of protein expression like alkaline phosphatase (ALP), a hepatic enzyme important in the diagnosis of disease [119]. Anticancer activity of fruit extract of *B. vulgaris* was also demonstrated on human breast cancer cells (MCF-7). The extract reduces cell proliferation in time and dose-dependent manner. It has also been evidenced the importance of the solvent for the extraction processes, because as it is reported in several studies, ethanol extract is more active than water extract, probably due to its capacity to extract more compounds responsible for anticancer activity like alkaloids [120]. *B. vulgaris* ethanol extract reduces viability in the breast, colon, hepatic and cervix cancer cell lines in a dose-dependent manner after incubation at 24, 48 and 72 h. The ethanol extract has a similar activity of berberine chloride [121]. This was also confirmed by El Khalki et al. [122], who studied cytotoxicity on human breast adenocarcinoma cell (MCF-7) of *B. vulgaris* and berberine.

The antioxidant activity might play a major role in increasing efficiency of such extracts to kill cancer cells and protect normal cells, besides the inhibition of cell growth. Choi et al. [123] demonstrated that treatment with berberine reduces p53 expression in the human prostate cancer cell. In fact, berberine promotes translocation of p53 in nuclei and arrest of the cell cycle in G0/G1. This was also confirmed in in vivo studies. In this sense, the intraperitoneal administration of berberine at 10 mg/kg caused a substantial decline in tumor volume and weight of prostate cancer. The effect is more evident in cancer expressing p53 (LNCaP) both in vivo and in vitro [123].

Berberine and curcumin have been tested on different types of cancer cell line models as A549 (lung cancer cell line), Hep-G2 (liver cancer cell line), MCF-7 (breast cancer cell line), Jurkat (leukemia cancer cell line) and K562 (kidney cancer cell line) by Balakrishna et al. [124]. This work can reveal the synergetic activity of these compounds. The anticancer effects in these cells are mediated by inducing apoptosis [124]. The synergistic effect of two different compounds was also evidenced by Ren et al. [125]. They showed as galangine and berberine together demonstrated an anticancer activity stronger than that showed when used singularly, on esophageal carcinoma cells. In fact, they induce apoptosis, promote cell cycle arrest in the G2/M phase and increase reactive oxygen species (ROS) in cancer cells [125]. The anticancer activity of *B. aristata* roots was also evaluated on human osteosarcoma cells [126].

*B. libanotica* root extract showed potential anti-inflammatory and anticancer activity, mostly due to alkaloids and other compounds. This effect was demonstrated on human colon cancer cells [127] in which berberine inhibits COX-2 transcriptional activity. *B. libanotica* extract reduced the viability of CD4 T-cells infected by the retrovirus HTLV1, a kind of cell characteristic of an aggressive form of leukaemia [128]. Moreover, root extract showed anticancer activity on different cell lines of prostate cancer; it reduces cell viability and promotes cell cycle arrest in G0/G1 [129]. Subsequent studies on human erythroleukemia cell lines investigated molecular pathways responsible for the anticancer activity. The extract induced apoptosis of cells through the modulation of Akt/NF-Kb/COX-2 signal transduction pathways [130]. *B. libanotica* extract showed a dominant effect on K562 cells by the activation of the late markers of apoptosis with caspase-3 activation, Poly (ADP-ribose) polymerase (PARP) cleavage and DNA fragmentation. The study demonstrated that treatment with the extract induces apoptosis in erythroleukemia cell line expressing COX-2 (HEL cells) or not (K562), especially at a dose of 300 μg/mL after 48h of treatment. In particular, *B. libanotica* extract induced activation of caspase-3 and -9, correlated with PARP cleavage and DNA fragmentation. In this process, the extract is more effective than berberine tested at a dose of 40 μg/mL. Moreover, the extract reduced significantly the expression of COX-2, which prevent apoptosis in cancer cells through the activation of Akt and NF-kB. A similar mechanism was shown for 4-chlorobenzoyl berbamine; and a synthetic compound derived from berbamine. It induces apoptosis in lymphoma cell lines and G2/M cell cycle arrest through PI3K/Akt and NF-kB signaling pathways [131]. Changes as esterification, etherification or sulfonylation on the structure of berbamine allowed to obtaining new molecules capable of solving the problem of resistant in many types of tumor [132]. Many synthetic derivatives of berbamine also demonstrated antineoplastic activity. Among these, BBMD3 was shown to be the most potent as an anticancer agent on human melanoma cells. It inhibits JAK/STA3 pathways reducing pro-apoptotic gene expression [133]. This was also confirmed in osteosarcoma and glioblastoma cell lines where BBMD3 induced inhibition of Jak2/STAT3 signaling pathway and activation of the stress response JNK pathway. Moreover, it increases the expression of miR-4284 involved in tumorigenesis and apoptosis [134,135].

Berbamine, which is contained in *B. amurensis,* has activity also tested on solid tumor. In fact, it arrests growth and migration in vitro and ex vivo of human lung cancer A549 cell line at low concentration trough down-regulation of anti-apoptotic protein Bcl-2 and up-regulation of the pro-apoptotic protein Bax [136]. *B. amurensis* extract arrests proliferation of Hepg2 and MCF-7 cells; extraction technique influences the presence of the active compound and consequently the extract activity [137].

*B. orthobotrys* is another species of this genus mainly grow in Iran. Roots bark leaves are used in traditional medicine in easy problem like menstrual pains, kidney stones, but the species have also demonstrated anticancer activity in HeLa cell line. As reported by Bavand et al. [138], ethanol root extract induces morphological change and apoptosis in HeLa cells then 72h of treatment. In particular treatment of cells with 1.25 mg/mL of extract, reduced the cell viability, inhibited the cell growth, changed cell adhesion to the substrate, pigmented the cells and formed apoptotic bodies [138]. Treatment with a low concentration of the extract also presents anticancer activity on other types of cancer cell line. Engel et al. [139] reported that the reduction of cell vitality of 60% caused by treatment with different doses ranged from 100 to 1 μg/mL of root extract of *B. orthobotrys.* They also studied the possible molecular mechanism responsible for cell death. Microscopic analysis of cells showed the accumulation of lysosome, which starts programmed cell death trough liberation of ROS and hydrolytic enzymes, granularization and formation of Golgi vesicles, as well as the diffuse distribution of neutral lipids. This is pronounced at 100 µg/mL, but also lower doses causes a slight formation of lysosome vesicles [139].

In addition to alkaloids there are other secondary metabolites with anticancer activity, for example, the triterpenoids the main active constituent of the trunk of *B. koreana*. They have been identified through mass spectrometry and nuclear magnetic resonance and tested in different cancer cell lines (A549, SK-OV-3, SK-MEL-2 and HCT-15) where they reduce cell proliferation [140,141].

## 7. Clinical Studies of *Berberis* Plants in Human

Currently available clinical trials regarding this group of plants point on their effects in various conditions related to cardiovascular diseases and associated risk factors, neurodegenerative diseases and inflammation.

One group of clinical trials conducting by Guiseppe Derosa et al. [142,143,144,145] had a specific interest in a fixed combination that included *B. aristata* and Silybum marianum (Berberol^®^). The reason for this combination lies in low bioavailability of *B. aristata*, while S. marianum is there to improve its intestinal absorption. A 52-week double-blind placebo-controlled study in 136 obese patients with type-2 diabetes mellitus (T2DM) and metabolic syndrome analyzed various parameters, including: Fasting blood glucose, insulin, total cholesterol, HDL, LDL, triglycerides, and body mass index (BMI). [146]. All of these parameters have been significantly improved in the treatment group compering with the baseline and in order to control group. Previously, comparing same fixed combination vs. *B. aristata* monotherapy in clinical trial conducting by Di Pierro et al. [147] with T2DM subjects, shown that combination is more effective in decreasing of HbA1c indicated that positive effects are partly due to S. marianum. In another study with the same combination of extracts in 102 dyslipidemia subjects after three months, it has been shown reducing of total cholesterol, triglycerides and LDL, with increasing of HDL from randomization and compared to the placebo group. The same result on lipid profile has been observed in a double-blind, randomized placebo-controlled trial that included 106 patients with metabolic syndrome treated with B. vulgaris [148]. Another two double-blind, randomized, placebo-controlled, 6-months clinical studies with Berberol^®^ conducting by Derosa et al. [142,143], followed dyslipidaemic subjects intolerant to statins at high dosages. In both studies were included patients tolerant to a half dose of statins. The lipid profile of included patients did not significantly change in the active treatment group after reduction of statins dosage and the introduction of Barberol^®^. Meanwhile, in placebo group lipid profile was worsened compared to baseline and with active treatment.

The clinical trial with type 1 diabetes mellitus (T1DM) subjects treated with the same fixed combination (Berberol^®^) showed decreasing of insulin dose necessary to reach adequate glycemic control [144]. The clinical trial with subjects at low cardiovascular risk also confirmed hypocholesterolemia effects of a fixed combination of Berberol^®^ [145].

Berberin has been shown to inhibit CYP3A4 in in vitro and animal models, as well as in humans, and that inhibition should increase blood levels of statins, cyclosporine, and calcium channel blockers, similar to the action of grapefruit [149]. The inhibition of enzyme CYP3A4 activity in humans has been observed in a two-phase randomized-crossover clinical study in healthy male subjects after two weeks of berberine administration (300 mg, p.o.) [150]. In a randomized double-blind placebo-controlled clinical trial, patients suffering from irritable bowel syndrome received berberine hydrochloride twice daily for two months [151]. The benefits from the treatment were observed as better IBS symptom and depression/anxiety scores.

One of the clinical trials examined the effect of aqueous extract of dried barberry taken orally as an anti-acne agent [152]. The results obtained from teenagers in this placebo-containing trial show the effectiveness of using barberry in the treatment of acne vulgaris, despite the treatment and control groups were small.

In all of these trials, no patients had serious adverse events. The limitations were the relatively small size of the sample, and relatively short follow-up period. However, the side effects of berberine have been reported in some in vitro and in vivo animal models, and observed effects were related to its neurotoxicity [153,154]. Despite that, in animal models of Alzheimer’s disease neuroprotective effects of berberine have been illustrated [155]. The published results in a review article from 2015 indicated that on web page www.clinicaltrials.gov there was 17 clinical trials on the efficacy of berberine [156], and currently there are 51 clinical trials which showing us increasing interest in beneficial effects of this compound [data obtained searching web page dated 24 May 2018].

## 8. Conclusions

*Berberis* is an important genus of wild plants with a multitude of uses in pharmacology and food industry. These species are the abundant source of important natural compounds, i.e., vitamins, minerals, alkaloids and antioxidants, which can be used in a wide array of pharmaceutical and nutraceutical products. Some of the species of the genus like *B. vulgaris* are also cultivated in Iran and other countries, but information regarding its cultivation, diseases and production technology is sparse. The present study has been carried out to report on the adaptation of different *Berberis* species, suitable agro-climatic conditions for the higher yield, its production technology, diseases and harvesting methods. However, further studies should be conducted to evaluate genetic diversity in the cultivated species for selection of high yielding genotypes, development of new varieties, yield enhancement through appropriate cultivation practices and integrated pest management (IPM) techniques.

Regarding food and pharmacological applications, *Berberis* is an important group of the plants having enormous potential in the food industry, and several reports of their antimicrobial activity have been found in the literature. Several phytochemicals found in fruits, leaves, stems and root have demonstrated biological activities. Phytochemicals present in EOs confer antimicrobial and antioxidant properties that make them useful as food preservatives. On the other hand, alkaloids present pharmacological properties, such as anticancer activities reported. However, not much information is available on the direct application of these plants in food products. On the other hand, the extracts of *B. vulgaris* were the most promising as antioxidants as well as inflammatory and neurological disorders protective. In addition, positive effects related to cancer targets have been reported to reduce cell proliferation without affecting a normal human cell. For this reason, *Berberis* spp. maybe considered an alternative for cancer treatment, but it is necessary to confirm their efficacy in vivo, especially investigating the toxicity during drug therapy.

## Figures and Tables

**Table 1:** Alkaloids from *Berberis* species.

Chemical structure	Name	Plant
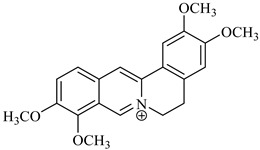	palmatine	*B. vulgaris*
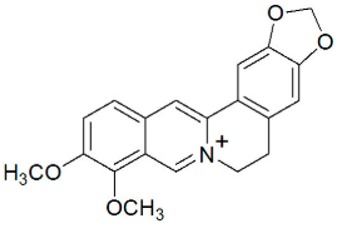	berberine	*B. vulgaris*
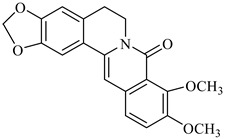	oxyberberine	*B. vulgaris*
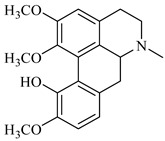	isocoridine	*B. vulgaris*
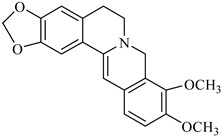	lambertine	*B. vulgaris*
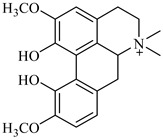	magniflorine	*B. vulgaris*
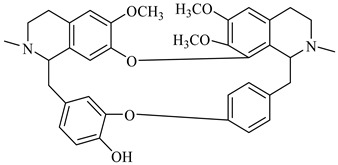	oxycanthine	*B. vulgaris*
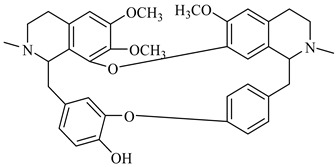	berbamine	*B. aristata*
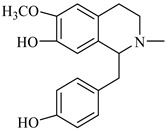	(+)-N-methylcoclaurine	*B. montana*
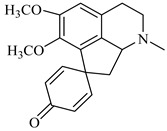	(−)-pronuciferine	*B. montana*
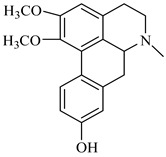	(+)-9-hydroxynuciferine	*B. montana*
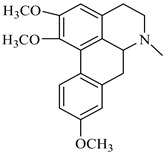	(+)-orientine	*B. montana*
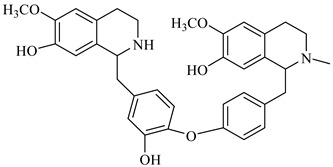	2-norberbamunine	*B. stoloniferais*
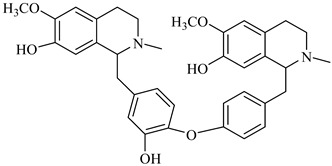	berbamunine	*B. stoloniferais*
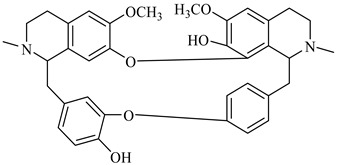	aromoline	*B. stoloniferais*
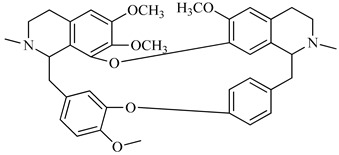	isotetrandrine	*B. stoloniferais*
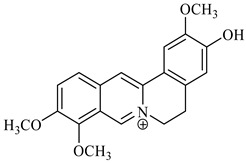	jatrorrhizine	*B. umbellate*

**Table 2:** A list of the antimicrobial potential of the *Berberis* species evaluated across the globe is provided which support the use of *Berberis* species in food preservation.

S. No.	Species	Part	Country	Extract/Model/Compound	Tested Micro-Organism	Results	Reference
1	*B. aristata*	Stem and leaves	Nepal	Hexane, Ethyl acetate, Methanol	*Staphylococcus aureus*, *Kleibsella pneumoniae*, *Salmonella typhimurium*	Against *S. aureus*: methanol significant zone of inhibition (21 mm), ethyl acetate extracts moderate activity, hexane extract of stem slightly active.	[73]
2	*B. aristata,* and *B. ligulata*	Bark stem Leaves	Nepal	Ethanol	*Bacillus subtilis*, *Escherichia coli*, *Pseudomona aeruginosa*, *Salmonella. typhi*, *Salmonella dyjenteriae*, *Salmonella cholerae*	Ethanol extract of *B. aristata:* largest zone of inhibition (21 mm) against *B. subtilis* and the smallest MBC value (90 mg/mL) for *S. aureus.* Gram positive bacteria more susceptible to the ethanol extract. *B. aristata* relatively broad-spectrum antibacterial activity.	[74]
3	B. vulgaris	Stem	Iran	Ethanol	*P. aeruginosa*, *Acinetobacter baumannii*, *E. coli* and *Salmonella enteritidis*	MIC determination: stem extracts inhibit the growth of all the studied bacteria (3900 to 37,500 μg/mL) by synergistic effects with ciprofloxacin.	[75]
4	*B. asiatica*	Leaves	Uttarakhand, India	Methanol	*E. coli, Enterobacter aerogenes, Proteus vulgaris, P. aeruginosa, K. pneumoniae, B. subtilis, S. aureus*	Methanol extracts of leaves: high inhibitory potential on *S. aureus, K. pneumoniae, E. coli, B. subtilis* and *P. vulgaris* in all concentration.	[76]
5	*B. aristata, B. asiatica, B. lycium*	Stem	Bangalore, India	Methanol	*Nocardia sp.*, *S*. *aureus*, *S. pneumonia*, *P. aeruginosa*, *Streptococcus viridians*, *E. coli*	Sensitivity to *Nocardia* sp., *S*. *pneumonia* and *E. coli*.	[77]
6	*B. glaucocarpa*	Root wood	Pakistan	Ethanol	SMRSA, EMRSA, *Mycobacterium marinum*, *E. coli*, *Trypanosoma brucei*	Berberine (MIC = 12.5 and 25 μg/mL), berberine chloroform (MIC = 25 and 12.5 μg/mL) and syringaresinol (12.5 μg/mL): very active against SMRSA, *M. marinum* and *T. brucei.*	[78]
7	*B. vulgaris*	Stem bark	Romania	Ethanol	*Botrytis cinerea*	*B*. *vulgaris* bark extract, berberine, and fluconazole significantly inhibited growth of *B. cinerea.*	[79]
8	*B. vulgaris*			Ethanol	*S. aureus*, *Staphylococcus epidermidis*, *K. pneumoniae*, *B. subtilis*, *E. coli*, *Aspergillus niger*, *Trichoderma*, *Alternaria solanai*	20 mm zone of inhibition against *E. coli.* Good activity against *B. Subtilis,* moderate against *Trichoderma,* insignificant against other stains.	[80]
9	*B. vulgaris* and its active constituent, berberine	Root	Egypt	Ethanolic extract	*Candida albicans*, *E. coli*	*Berberis* ethanolic extract and berberine standard can inhibit *C. albicans* and *E. coli* growth.	[81]
10	*B. vulgaris*	Fruit	Pakistan	Distilled water	*S. aureus*, *Proteus*, *S. typhi, Salmonella paratyphi A*, *Salmonella paratyphi B, K. pneumoniae*, *E. coli*, *P. aeruginosa*	Antibacterial activity against all tested pathogens.	[82]
11	*B. thunbergii*	Fruit	Hungary	Juice; water extract and -methanol extract	*B. subtilis*, *Bacillus cereus* var. *mycoides*, *E. coli, Serratia marcescens*	Juice, water extract and methanol extract showed activity against all bacteria.	[83]
12	*B. calliobotrys*	Stems and branches	Pakistan	Methanol	*B. subtilis*, *P. aeruginosa*, *S. aureus* fungal strains namely *C. albicans*, *Penicillium notatum*	The methanol extract, ethyl acetate and n-butanol fractions: maximum zone of inhibition against all bacterial strains especially *S. aureus* and antifungal effects.	[84]
*13*	*B. lycium*	Roots	Libya	Distilled water, ethanol, isopropanol and methanol	*Pseudomonas* sp., *E. coli*, *Streptococcus* sp., *Staphylococcus* sp.	Methanolic displayed maximum inhibitory zone (16 mm), isopropanol extract (13 mm) and ethanol extract (12 mm). The aqueous extract exhibited the least inhibitory zone (10 mm). The methanolic extract: maximum inhibitory zone (12 mm), *Pseudomonas* (11 mm) and *Staphylococcus* (10 mm).	[85]
14	*B. hispanica*	Root Bark	Marocco	Ethanolic extract	*Mycobactérium smegmatis, Mycobacterium aurum*	The ethanolic extract from root bark displayed an important antimycobacterial activity. The inhibition zones for *M. aurum A+* were significantly larger than those for *M. smegmatis* MC2.	[86]
15	*B. ruscifolia*	-	Argentina	Acetone, chloroform-methanol (1:1) and methanol	*E. coli*, *P. aeruginosa*, *Listeria monocytogenes*, *S. aureus*	All extracts exhibited antibacterial activity with MIC varying from 16 to 2 mg/mL. The highest inhibition with acetonic and chloroform-methanolic extracts of species against *S. aureus* (MIC = 2 mg/mL). Methanolic extracts *B. ruscifolia* showed no antibacterial activity against all tested bacteria.	[87]
16	*B. aristata*	Stem bark	India	Ethanol and aqueous extracts	*Shigella flexneri*, *Shigella sonnei*, *Shigella dysenteriae*, *Shigella boydii*	Extracts of *B. aristata*: antibacterial activity against four strains of *Shigella* (8 and 23 mm).	[88]
17	*B. aristata, B. asiatica, B. chitria* and *B. lycium*	Root and stem	India	Ethanol	*Micrococcus luteus*, *B. subtilis*, *B. cereus*, *Enterobacter aerogenus*, *E. coli*, *K. pneumoniae*, *Proteus mirabilis*, *P. aeruginosa*, *S. aureus, S. typhimurium*, *Streptococcus pneumonia*, Fungal strains *Aspergillus nidulans*, *C. albicans*, *Aspergillus terreus*, *Trichophyton rubrum, Cistus albidus*, *Aspergillus flavus*, *A. niger*	*B. lycium*, *B. aristata* and *B. asiatica* root extract showed significant antifungal activity against *A. terreus* and *A. flavus*. *B. aristata* root and *B. lycium* (stem) extracts gave very low MIC values (0.31 μg/mL) as compared to other tested species.	[89]
18	*B. Lycium*	Root	Pakistan	Ethanol, petroleum ether	*S. aureus*, *S. epidermidis*, *B. subtilis*, *S. typhi, E. coli, C. albicans*	The ethanolic and aqueous crud root extract: most effective antifungal and antibacterial agents.	[90]
19	*B. integerrima* Syn: *B. densiflora*	Roots	Iran	Methanol	*Brucella abortus*	MIC and MBC results, jatrorhizine exhibited higher antibacterial activity with MIC (0.78 μg/mL) and MBC (1.56 μg/mL) compared with the standard (streptomycin, 10 μg/mL).	[91]
20	*B. lycium*	Roots	Pakistan	Hydric extract	*E. coli*, *Pseudomonas*, *Staphylococcus*, *Proteus*	Significant activity against *E. coli* and Proteus (80 to 100%), while it demonstrated a good activity against *Pseudomonas* and *Staphylococcus* (60 to 70%).	[92]
21	*B. aristata*	Bark and leaves	India	Methanol, ethanol and hexane	*B. subtilis, Agrobacterium tumefaciens*, *E. coli*, *Xanthomonas. Phaseoli, Erwinia chrysanthemi*	All the extracts of tested plants showed variable activity against all the tested bacterial strains. Methanol extract revealed highest antibacterial activity (11 mm) recorded against *E. chrysanthemi*. Hexane extract: totally inactive against all the tested strains.	[93]
22	*B. aristata*	Roots	India	Aqueous and alcohol extracts	*S. aureus*, *B. subtilis*, *E. coli, S. typhimurium*	Alcoholic and aqueous extract showed antimicrobial activity against four tested bacteria. *B. aristata* exhibited highest zone of inhibition for *B. subtilis* followed by *S. aureus*, *E. coli* and *S. typhimurium.*	[94]
23	*B. microphylla*	Leaves, stems and roots	Chile	Methanol	*E. coli*, *S. typhimurium*, *L. monocytogenes*, *E. aerogenes*, *S. aureus, B. cereus*, *S. epidermidis* and *B. subtilis*	All extract possesses significant antibacterial activity against Gram-positive bacteria but not against Gram-negative bacteria.	[95]
24	*B. lycium*	Root bark	Pakistan		*E. coli*, *K. pneumoniae*, *P. aeruginosa*, *S. aureus*, *B. subtilis*	Silver nanoparticles were very active against Gram-negative and Gram-positive bacteria Aqueous bark extract (10 μg/mL) possess highest activity against *E. coli* and *P. aeruginosa.*	[96]
25	*B. vulgaris*	Fruit	Iran		*L. monocytogenes*	Average diagonal of growing area in disk diffusion test for species: 12 mm and MIC was 125 μg/mL and MBC of *B. vulgaris* was 500 μg/mL.	[97]
26	*B. aristata*	Stem bark	Alcohol	In vivo in an animal model using Sprague Dawley rats	Carbapenem-resistant *E. coli*	An aquo-alcoholic extract of the species: effectively manage peritonitis induced by Carbapenem-resistant *E. coli* in a rat model at a single post-exposure prophylactic dose of 0.5 mg/kg body weight.	[98]
27	*B. aristata*	Roots	India	Aqueous and alcoholic extract of fresh roots, as well as aqueous extract of dried roots	*S. aureus*, *S. epidermidis*, *Streptococcus pyogenes*, *Streptococcus viridans*, *Enterococcus faecalis*, *B. subtilis*, *B. cereus*, *E. coli*, *K. pneumoniae*, *P. aeruginosa*, *P. vulgaris*, *P. mirabilis*, *S. typhi*, *S. paratyphi A*, *S. typhimurium*, *S. dysenteriae* type 1, *Vibrio cholerae*	All three extracts displayed wide antibacterial activity against Gram-positive bacteria. Among the Gram-negative bacteria tested, the antibacterial activity was limited to *E. coli*, *S. typhimurium, S. dysenteriae* type 1 and *V. cholerae*. All extracts also possess antifungal activity against the fungal species tested, except *Candida krusei*.	[99]
28	*B. aristata*	Root Stem Leaf	Pakistan		*E. coli*, *S. typhi*, *S. aureus*, *Shigella, Citrobacter*, *P. vulgaris*,*Enterobacter, Streptococcus pyrogenes*, *V. cholera, Klebsiella* spp., *A. niger*, *Cladosporium*, *Rhizoctonia*, *Alternaria*, *Trichoderma*, *Penicillium*, *Curvularia*, *Paecilomyces* and *Rhizopus*	The extracts significantly inhibited the growth of the studied microbes, except *A. niger*, *Curvularia*, *Paecilomyces* and *Rhizopus*.	[100]
29	*B. aristata*		India		*V. cholerae*, *S. aureus*	All the strains of *V. cholerae* are susceptible. All the *Salmonella* sp., *Pseudomonas* sp., and some of the E. *coli* strains are highly resistant, except some strains of *E. coli* as AL26, and *Shigella* sp. are susceptible. All *Xanthomonas* sp. were highly susceptible. Berberine sulfate showed antifungal action against *C. albicans*, *Candida tropicalis*, *Trichophyton mentagrophytes*, *Microsporum gypseum*, *Cryptococcus neoformans* and *Sporothrix schenkii, Mycobacterium tuberculosis* var. *hominis* H_37_RV and *Entamoeba histolytica.*	[101]
30	*B. heterophylla*	Leaves, stems and roots berberine	Argentina		*S. aureus, E. faecali, P.aeruginosa, E. coli, C. albicans, Candida glabrata, Candida haemulonii, Candida lusitaniae, C. krusei, Candida parapsilosis*	The aqueous extracts of *B. heterophylla* do not possess significant antimicrobial activity. Berberine displayed a significant antibacterial and antifungal activity against *S. aureus* and different *Candida* spp., some of them obtained from the clinical isolated.	[102]
31	*B. amurensis*	Branches and leaves	Korea		*Bacillus atrophaeus*, *Kocuria rhizophila*, *M. luteus*, *S. epidermidis*, *B. subtilis subsp. Spizizenii*, *K. pneumoniae*, *Enterobacter cloacae*, *Salmonella enterica subsp. enterica, P. aeruginosa*	No significant activity against gram-negative bacteria.	[103]
32	*B. croatica* and *B. vulgaris*	Roots, leaves, and twigs	Croatia	Ethanol	*B. subtilis*, *S. aureus*, *E. coli*, *P. aeruginosa*, *C. albicans*	Extracts of both species: significant antibacterial activity against the Gram-positive bacteria. Root extracts of *B. croatica:* activity against *P. aeruginosa*, and leaf extracts against *B. subtilis*. Neither species possessed antifungal activity. Leaf extracts of *B. croatica*: antibacterial activity against *B. subtilis*. Likewise, neither of the species extracts showed activity against *E. coli* and *C. albicans*, except when were diluted. Ethanolic extracts of twigs of both species: inactive against *B. subtilis* and against *S. aureus,* with the exception of *B. croatica* twig from Kiza locality.	[104]
33	*B. lycium*	Roots	India	Hexane extract, Methanolic extract, aqueous extract and berberine	*K. pneumonia, E. coli, P. aeuroginosa*, *S. aureus*, *B. subtilis*, *C. albicans*, *A. niger*, *Aspergillus fumigates*	Methanolic extract of species was highly effective against *E. coli*, *S. aureus*, *B. subtilis*, *C. albicans*, *A. fumigates*. Pure berberine was effective against *E. coli* and *C. albicans*.	[105]
34	*B. aetnensis*	Roots	Italy	Ethanol ether and chloroform	*S. aureus*, *B. subtilis*, *E. faecalis*, *E. coli*, *P. aeruginosa*, *Stenotrophomonas maltophilia*, against 14 strains of nosocomial origin: two strains of *S. aureus* (1 Met-S, 1 Met-R); four strains of *S. epidermidis* (2 Met-S, 2 Met-R); three strains of *E. coli*; four strains of *P. aeruginosa*, *Hafnia alvei* and *C. albicans*, *C. parapsilosis*, *C. krusei*	The root and leaf extracts showed a greater activity against Gram-positive bacteria and yeasts than against Gram-negative bacteria, except for *P. aeruginosa*. The chloroform extract of leaves was more active than the ethanol.	[106]
35	*B. thunbergii, B. vulgaris*	Roots	USA		*E. coli*, *P. aeruginosa*, *S. aureus*, *S. mutans*, and *S. pyogenes*	Ethanolic extracts more active against studied bacteria, strongest activity against *S. pyogenes* and *S. aureus*.	[107]
36	*B. vulgaris*	Root bark	Algeria	Methanol and water	*S. aureus*, *E. faecalis*, *E. coli*, *E. cloacae*, *K. pneumoniae*, *P. aeruginosa*	The extracts of species root barks presented a strong activity against *S. aureus* (23.0 mm), a weak activity against *E. faecalis* (13.0 mm) and no activity toward other strains.	[108]
